# Severe Acute Respiratory Syndrome Coronavirus 2 Receptor (Human Angiotensin-Converting Enzyme 2) Binding Inhibition Assay: A Rapid, High-Throughput Assay Useful for Vaccine Immunogenicity Evaluation

**DOI:** 10.3390/microorganisms11020368

**Published:** 2023-02-01

**Authors:** Joyce S. Plested, Mingzhu Zhu, Shane Cloney-Clark, Edmond Massuda, Urvashi Patel, Andrew Klindworth, Michael J. Massare, Rongman Cai, Louis Fries, Greg Glenn, Raj Kalkeri

**Affiliations:** 1 Clinical Immunology, Novavax, Gaithersburg, MD 20878, USA; 2 Discovery, Novavax, Gaithersburg, MD 20878, USA; 3 Biostatistics, Novavax, Gaithersburg, MD 20878, USA

**Keywords:** COVID-19, human angiotensin-converting enzyme 2 (hACE2), immunogenicity, SARS-CoV-2, assay validation, neutralizing antibody titers, correlate of protection, immune evasion

## Abstract

Emerging variants of severe acute respiratory syndrome coronavirus 2 (SARS-CoV-2) show immune evasion of vaccine-derived immunity, highlighting the need for better clinical immunogenicity biomarkers. To address this need, an enzyme-linked immunosorbent assay-based, human angiotensin-converting enzyme 2 (hACE2) binding inhibition assay was developed to measure antibodies against the ancestral strain of SARS-CoV-2 and was validated for precision, specificity, linearity, and other parameters. This assay measures the inhibition of SARS-CoV-2 spike (S) protein binding to the receptor, hACE2, by serum from vaccine clinical trials. Inter- and intra-assay precision, specificity, linearity, lower limit of quantitation, and assay robustness parameters successfully met the acceptance criteria. Heme and lipid matrix effects showed minimal interference on the assay. Samples were stable for testing in the assay even with 8 freeze/thaws and up to 24 months in −80 °C storage. The assay was also adapted for variants (Delta and Omicron BA.1/BA.5), with similar validation results. The hACE2 assay showed significant correlation with anti-recombinant S immunoglobulin G levels and neutralizing antibody titers. This assay provides a rapid, high-throughput option to evaluate vaccine immunogenicity. Along with other clinical biomarkers, it can provide valuable insights into immune evasion and correlates of protection and enable vaccine development against emerging COVID-19 variants.

## 1. Introduction

Severe acute respiratory syndrome coronavirus 2 (SARS-CoV-2) is a coronavirus that causes the coronavirus disease 2019 (COVID-19), which was declared a pandemic illness starting in 2020. As of 2022, the pandemic is still ongoing despite vaccination, testing, and quarantining efforts worldwide [1]. This is in part because of the emergence of SARS-CoV-2 variants, some of which escape disease convalescent or vaccine-driven immunity. Some variants of concern, including Alpha, Beta, Gamma, Delta, and Omicron, have been associated with greater transmissibility, greater disease severity, or decreased responses to vaccines [2,3,4].

Identification of reliable, precise, specific, sensitive, and easily measured biomarkers of immunogenicity is critical for assessment of any vaccine. This presents a major challenge in fast-paced vaccine development, especially given the diversity of vaccine types, emerging viruses, and pathogenic mechanisms [5]. Given the relatively short time that COVID-19 has been a public health concern, biomarkers are still being identified to assess immunogenicity and estimate vaccine efficacy in real time. Some of the current biomarkers used to assess vaccine immunogenicity are anti-spike (S) or anti-receptor binding domain (RBD) immunoglobulin G (IgG) antibodies [2,5,6,7,8], neutralizing antibody responses [2,5,6,7,8,9], and levels of activated T cells [2,5]. Each of these markers has limitations. Neither anti-S nor anti-RBD IgG binding directly measure neutralization function of the antibodies. Neutralizing antibodies measured by microneutralization assays may block infection but may not be specific to a certain target or be homogenous. Higher counts of CD4+/CD8+ T cells specific for SARS-CoV-2 epitopes may be associated with reduced severity of infection but are more difficult to measure, and their role in COVID-19 vaccine immunogenicity is still unclear [2]. Additionally, emerging variants of SARS-CoV-2 such as Omicron have shown differences in reliability as correlates of protection [2]. An improved assay to measure immunogenicity of vaccines while overcoming the limitations of these current biomarkers is necessary.

SARS-CoV-2 infects cells by binding of viral S-protein to the receptor, human angiotensin-converting enzyme 2 (hACE2), on the target cell [10]. Results of several studies have demonstrated that blocking this binding process can prevent infection by SARS-CoV-2 [11,12,13,14], and hACE2 binding is widely thought to be a promising target for future COVID-19 therapeutics [15,16]. This article describes the development of an in vitro assay to detect inhibition of S-protein binding to hACE2 (most likely by anti-S antibodies) for the ancestral strain of SARS-CoV-2 and the Delta and Omicron BA.1/BA.5 variants. Additionally, the hACE2 binding inhibition assay was tested for precision, specificity, linearity, and other parameters. The hACE2 binding inhibition assay was found to provide a rapid, high-throughput option for evaluating vaccine immunogenicity, and results from the assay correlate well with other immunological markers such as anti-recombinant spike (anti-rS) IgG and neutralizing antibodies. The assay overcomes some limitations of previous vaccine immunogenicity assays by specifically detecting inhibition of hACE2 binding by SARS-CoV-2 S-protein, a critical step in SARS-CoV-2 infection.

## 2. Materials and Methods

### 2.1. Assay Procedure

**Figure 1:**
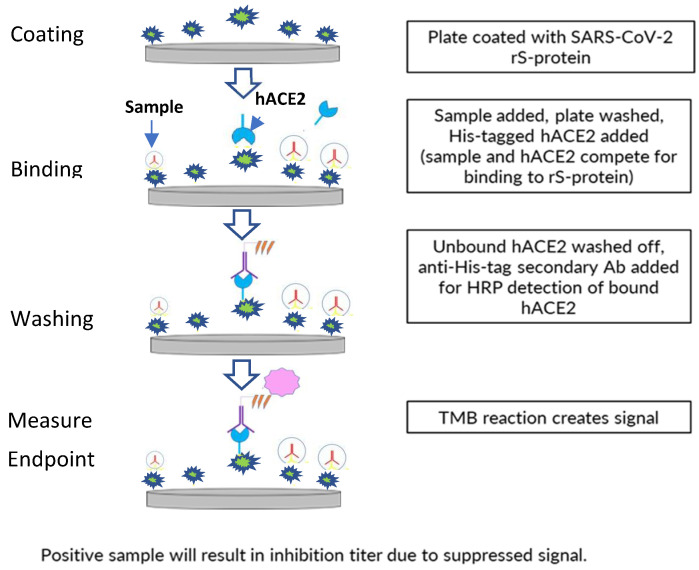
Procedure for performing the hACE2 binding inhibition assay. Ab—antibody; hACE2—human angiotensin-converting enzyme 2; His— polyhistidine; rS—recombinant spike; SARS-CoV-2—severe acute respiratory syndrome coronavirus 2; TMB—3,3′5,5′-tetramethylbenzidine.

A 96-well plate format, enzyme-linked immunosorbent assay-based assay was developed to assess inhibition of binding of ancestral strain or variant (Delta, Omicron BA.1/BA.5) SARS-CoV-2 S-protein (full-length wild-type rS protein with transmembrane domain) to the receptor hACE2 by human sera from clinical trials of the NVX-CoV2373 vaccine (Figure 1).

The 96-well assay plates (Thermo Fisher Scientific, Waltham, MA, USA) were coated with 0.8 µg/mL of SARS-CoV-2 rS-protein (produced at the Novavax, Inc., Gaithersburg, MD, USA) using a standard plate-coating method overnight at 2–8 °C. This was followed by washing with phosphate-buffered saline with Tween 20 (PBST) and blocking with blocking buffer (Thermo Fisher Scientific) for 1 h. Diluted serum samples were then added to the plate (60 min. of incubation), followed by 4 washes with PBST and the addition of polyhistidine-tagged hACE2 (from Sino Biologicals; approximately 30 ng/mL, depending on lot activity).

After unbound hACE2 was washed away with PBST, bound hACE2 was detected by incubating the plate with anti–polyhistidine-tagged/horseradish peroxidase secondary antibodies at room temperature (RT) for 1 h, then washing with PBST, followed by incubation with 3,3′5,5′-tetramethylbenzidine substrate (Thermo Fisher Scientific) for 30 min. Quantitation of positive 3,3′5,5′-tetramethylbenzidine reaction signal indicates the level of inhibition of hACE2 binding by serum components, as the amount of bound hACE2 giving the signal is inversely proportional to the amount of hACE2-binding inhibitors in the serum. The 50% inhibitory titer was calculated using a 4-parameter logistical fit and was compared with hACE2 controls. The percent hACE2 binding inhibition was calculated at all dilution points based on the hACE2 absorbance (optical density, OD), background absorbance (OD), and absorbance (OD) at each dilution point. The reciprocal dilution at which the serum inhibits hACE2 binding to S protein by 50% based on background-adjusted OD was reported as the hACE2 inhibition titer of the serum sample.

### 2.2. Samples

Healthy human serum samples collected in 2018 before the COVID-19 pandemic were obtained from commercial sources (BioIVT in Westbury, NY, USA; *n* = 13). Human convalescent serum samples (harvested during the COVID-19 pandemic or from people vaccinated as part of the NVX-CoV2373 vaccine trials) were from the Novavax clinical sample repository. For selectivity analyses, serum samples from a Novavax influenza vaccine trial were used. Positive quality control (QC) samples (COVID-19 convalescent serum pools) known to have high QC, mid QC, or low QC hACE2 inhibition titers were used. Negative controls were pre-pandemic sera negative for hACE2 binding inhibition in the assay. QC samples were tested in duplicate wells on the first plate of each run. For correlation analyses, serum samples were from the Novavax clinical trials 2019nCoV-101 (NCT04368988) and PREVENT-19 (NCT04611802).

### 2.3. Validation Assays

#### 2.3.1. Precision

Twenty samples were tested twice in an assay run (total of 6 runs by 2 analysts on 3 days), and each sample was tested in duplicate, with geometric mean titer (GMT) of duplicate values considered the inhibition titer. Precision was then estimated by calculating percent geometric coefficient of variation (%GCV) based on variance component analysis using sample as a fixed effect and analyst and day as the random effects.

Target precision was such that at least 80% of samples have a %GCV ≤20%, and %GCV ≤25% for samples at lower limit of quantitation (LLoQ).

#### 2.3.2. Specificity

hACE2 binding inhibition-positive serum samples were incubated with rS protein at RT before testing (5 samples). Controls used to set baseline were the same samples incubated with assay buffer only. The irrelevant nonspecific protein group used the same samples but incubated with respiratory syncytial virus (RSV) F protein or Ebola glycoprotein (GP), produced using same recombinant protein platform as for rS protein. Samples were then tested in the assay and inhibition titers were compared, with % reduction calculated as follows:

(1) $$\%\;\mathrm{Reduction}=\left[100-\frac{\mathrm{Results}\;\mathrm{with}\;\mathrm{or}\;\mathrm{without}\;S\;\mathrm{protein}\;\mathrm{incubation}}{\mathrm{Results}\;\mathrm{without}\;S\;\mathrm{protein}\;\mathrm{incubation}}\times 100\right]$$

#### 2.3.3. Selectivity

Nineteen samples collected before the COVID-19 pandemic (assumed to be negative for SARS-CoV-2 antibodies) and expected to be below the LLoQ were tested for hACE2 binding inhibition titers. Some samples were also tested for influenza hemagglutination inhibition (HAI) titers to assess whether varying levels of HAI titers interfered with detection of hACE2 binding inhibition titers [17].

#### 2.3.4. Linearity

Two hACE2 binding inhibition–positive samples with mid to high titer were tested in the assay undiluted or in a 1:2 dilution series of 10 dilutions (5 assays by different analysts), precision and accuracy of titer were calculated at each dilution point, and linear regression was conducted for observed versus expected GMT. The expected titer at each dilution was calculated from the overall GMT from all runs of the least diluted sample divided by the dilution factor, and observed GMT was the overall GMT from all runs. The % relative bias at each dilution point was calculated as follows:

(2) $$\%\;\mathrm{Relative}\;\mathrm{Bias}=100\times \frac{\begin{array}{c} \mathrm{Observed}\;\mathrm{overall}\;\mathrm{hACE}2\;\\ \mathrm{binding}\;\mathrm{inhibition}\;\mathrm{GMT} \end{array}-\begin{array}{c} \mathrm{Expected}\;\mathrm{hACE}2\;\\ \mathrm{binding}\;\mathrm{inhibition}\;\mathrm{GMT} \end{array}}{\mathrm{Expected}\;\mathrm{hACE}2\;\mathrm{binding}\;\mathrm{inhibition}\;\mathrm{GMT}}\;$$

#### 2.3.5. Sensitivity

The lowest (LLoQ) titer values that were accurately and precisely determined were assessed for the 2 samples in the linearity analysis. The LLoQ for the assay was set at 10 based on prior data accumulated with convalescent sera from COVID-19 cases; data developed here in both precision and dilutional linearity experiments confirmed the LLoQ based on acceptable GCVs obtained for samples and dilutions with results <15.

#### 2.3.6. Assay Robustness (Incubation Time)

Incubation time robustness—The assay was conducted using upper and lower incubation time limits for each incubation step; then, results were compared with the 6 runs conducted for the precision analysis (reference condition).

The target was such that ≥80% of samples should have values within ±20% of the reference (80–120% of reference values). Percent recovery and percent difference were calculated as follows:

(3) $$\%\;\mathrm{Recovery}=100\times \frac{\mathrm{Test}\;\mathrm{value}}{\mathrm{Reference}\;\mathrm{value}}\;$$

(4) % Difference=% Recovery −100 

#### 2.3.7. Sample Stability

Samples were stored at various temperatures (6 or 24 h at RT [*n* = 16], 7 or 14 days in 2–8 °C [*n* = 15], 9 or 24 months at –80 ± 10 °C [*n* = 16]), then tested in the assay, and the results were compared with freshly thawed samples (RT/refrigerator) or the original precision results (freezer at –80 °C). Samples at RT were thawed and then put in a 24 ± 2 °C incubator for 6 or 24 h. The samples stored at –80 °C were aliquots of the original samples from the precision assay. Samples were also tested after undergoing 7 or 8 freeze/thaw cycles (1 h at RT followed by refreezing), and results were compared with aliquots of the same samples that underwent only 1 freeze/thaw cycle. Percent recovery was calculated for all samples, as shown above. Only convalescent serum was used, as clinical trial samples were not available at the onset of testing.

#### 2.3.8. Matrix Effects

For hemolysis analyses, hemolyzed human serum was spiked into 5 samples and a negative control sample to create 50% hemolyzed or 25% hemolyzed samples (representing severe hemolysis). The samples were then tested in the assay, and percent recovery was calculated as shown above and compared with normal samples without hemolysis.

For lipemia analyses, serum with high levels of triglycerides was spiked into 6 samples to create samples with a final triglyceride concentration of 500 or 250 mg/dL (normal level, <150 mg/dL). The samples were then tested in the assay, and percent recovery was calculated, as shown above and compared with normal samples without lipemia.

### 2.4. Variant Assays

The assay validation method and protocol for the variants (Delta, Omicron BA.1/BA.5) followed a similar experimental, qualification, and assay validation plan as for ancestral strain, but the S protein coated onto the plate was replaced with proteins reflecting the respective variant sequences. Assay precision, dilution linearity, assay specificity, selectivity, LLoQ/upper level of quantification (ULoQ), and assay robustness (coating time) were assessed for each variant. Matrix interference, assay robustness (incubation times), and sample stability were assessed as part of the validation process for the original ancestral strain assay.

### 2.5. Correlation Analyses

For each sample, detection of anti-rS IgG antibodies and microneutralization antibodies was performed using methods previously described [18], then results were compared with hACE2 binding inhibition assay titers, and linear regression analysis was performed using GraphPad Prism software (San Diego, CA, USA; Version 9.3.1). For some serum samples, hACE2 binding inhibition assay results for prototype and Omicron BA.1 were observed at limit of detection (LOD) levels, most probably because of low levels of hACE2 binding inhibition. To avoid the effect of these samples on the correlations, correlation analysis was performed with and without including the hACE2 binding inhibition results at LOD. As there was no significant difference in the strength of correlations, plots of hACE2 binding inhibition versus anti-rS IgG levels for prototype and Omicron BA.1 excluding the data points at LOD are shown in the main text. Correlations for all the data points are shown in the supplement.

## 3. Results

### 3.1. Ancestral Strain Assay Validation Parameters

Inter-assay and intra-assay precision was <20% GCV for all 20 convalescent serum samples available at the time of assay development. Total assay precision for 90% of samples (18/20) was <20% GCV, meeting acceptance criteria (Table 1). Later, 21 serum samples from vaccinated individuals also showed <20% GCV and represented a wide range of titers.

Assay specificity met the acceptance criteria. All tested samples showed at least 74.1% reduction in hACE2 binding inhibition titers after pre-incubation of sera with SARS-CoV-2 rS-protein (≥50% reduction needed for acceptance). The samples also showed less than 20% change in inhibitory titer for irrelevant protein, when incubated with RSV F protein or Ebola GP (Table 2).

Assay selectivity met the acceptance criteria, as all samples collected before the pandemic showed negative (<LLoQ) results for hACE2 binding inhibition. Paired pre/post (day 0/day 21) samples from participants showing strong responses to influenza immunization showed that detection was not affected by large vaccine-induced changes in HAI titers (Table 2).

**Figure 2:**
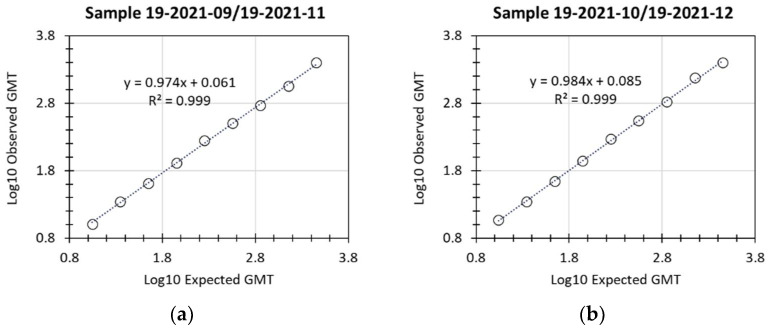
hACE2 binding inhibition assay ancestral strain linearity. Two hACE2 binding inhibition-positive samples were tested undiluted or diluted in 1:2 series in 5 assay runs by 3 analysts. Linearity was then evaluated by calculating precision and accuracy of the titer at each dilution as well as the slope of linear regression lines for each sample. For the 2 different samples, (**a**,**b**), dilutions are shown from undiluted to 1:256 dilution.

Linearity of the assay was successfully demonstrated, with R^2^ values of 0.999 for both of the 2 individual samples examined (Figure 2 and Table 3). LloQ was assigned a titer of 10, based on the lowest titer values that were accurately and precisely detected for the 2 samples (expected titer of 8.3 or of 13.9). UloQ was determined to be at least 2540.1 based on the highest hACE2 binding inhibition titers from the linearity analysis and based on clinical samples available at the time of validation of this assay. With the availability of additional samples from clinical trials, the accumulation of sera with titers greater than the current UloQ is expected. At regular time intervals, the precision and dilutional linearity of the assay for any such sera will be re-examined, and the UloQ updated.

There was minimal impact on the assay by the presence of free hemoglobin or a lipemic matrix (Table 4).

**Figure 3:**
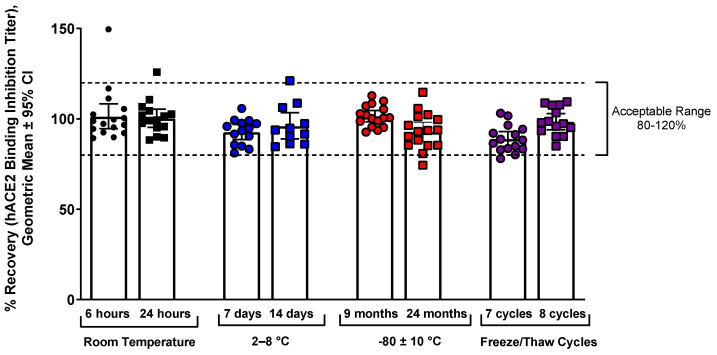
hACE2 binding inhibition assay ancestral strain temperature and freeze/thaw stability. Samples were stored at various conditions (RT, refrigeration, or freezing) for various lengths of time, or were subjected to multiple freeze/thaw cycles, then were used in the hACE2 binding inhibition assay to determine sample stability. The acceptable range of deviation was 80% to 120% of the reference condition values (dashed lines).

Stability of samples was tested at RT, refrigeration (2–8 °C), and freezing (−80 °C), and the samples were stable up to 8 freeze/thaw cycles and up to 24 months in freezer storage (Figure 3).

Assay robustness for incubation time was demonstrated for the ancestral strain, as 95% of samples at the lower limit of incubation time and 85% of samples at the upper limit of incubation time had hACE2 binding inhibition GMT between 80% to 120% of the reference condition values (Tables S1 and S2).

### 3.2. Variant Strain Assay Validation

**Figure 4:**
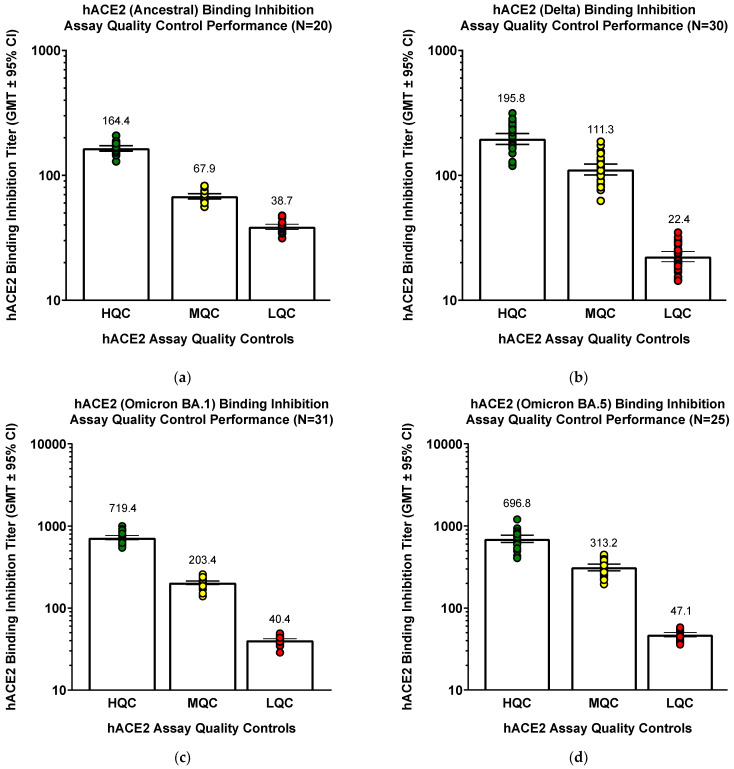
hACE2 binding inhibition assay ancestral strain and variants quality control: performance of standards. (**a**) For ancestral strain, (**b**) Delta variant, (**c**) Omicron BA.1 variant, or (**d**) Omicron BA.5 variant. QC samples were tested in the hACE2 binding inhibition assay to examine assay performance. For ancestral strain, HQC should have a titer between 107.3 and 223.7, MQC should be between 46.3 and 90.3, and LQC should be between 25.7 and 52.2 (inclusive). For Delta variant, HQC should have a titer between 114.5 and 334.9, MQC should be between 64.8 and 191.0, and LQC should be between 13.6 and 36.8 (inclusive). For Omicron BA.1 variant, HQC should have a titer between 445.7 and 1161.2, MQC should be between 138.6 and 298.4, and LQC should be between 28.8 and 56.7 (inclusive). For Omicron BA.5 variant, HQC should have a titer between 426.9 and 1137.4, MQC should be between 201.0 and 487.9, and LQC should be between 34.8 and 63.6 (inclusive). GMT values are shown above each bar.

The original assay was developed using ancestral strain, but controls (different sets of QC serum controls for each strain) performed similarly well even when the assay was modified for Delta and Omicron BA.1/BA.5 variants (Figure 4). QC samples (COVID-19 convalescent serum pools) known to have high QC, mid QC, or low QC hACE2 inhibition titers were used.

Similar results for the validation parameters were seen for Delta and Omicron BA.1 variants. For Delta variant, 16 of 21 samples (76.2%) had intra-assay, inter-assay, and total hACE2 binding inhibition titer %GCV <30% (total assay precision for all 21 samples was 24.1% GCV). Although these results seem to be slightly less precise than for ancestral strain, it is possible that antibodies elicited with an ancestral strain vaccine might have lower (or to be accurate, more variable) affinity for Delta S, and this could be a contributor to a slight reduction in precision for Delta hACE2 binding inhibition.

Incubation with SARS-CoV-2 rS protein before the assay decreased hACE2 binding inhibition titer in 5 of 6 samples, whereas only 1 of 6 (RSV F protein) or 2 of 6 (Ebola GP) sample were affected by irrelevant proteins. Linearity was successfully demonstrated (R^2^ = 0.996), LLoQ was defined as 10, and ULoQ was determined to be at least 2540.1. Robustness for incubation time was demonstrated, as 22 samples (81.8%) at the lower limit of coating time (14 h) and 19 samples (100%) at the upper limit (48 h) had hACE2 binding inhibition GMTs of 70% to 130% of reference condition values.

For Omicron BA.1 variant, 20 of 23 samples (87.0%) had inter-assay and total hACE2 binding inhibition titer % GCV <20% (total assay precision for all 21 samples was 13.3% GCV). For intra-assay precision, 22 of 23 samples (95.7%) had % GCV <20%. Incubation with SARS-CoV-2 rS protein before the assay decreased hACE2 binding inhibition titer in 4 of 6 samples, whereas none of the samples were affected by irrelevant proteins. Linearity was successfully demonstrated (R^2^ = 0.988), LLoQ was defined as 10, and ULoQ was determined to be at least 2540.1. Robustness for incubation time was demonstrated, as 19 samples (86.4%) at the lower limit of coating time (14 h) and 18 samples (85.7%) at the upper limit (48 h) had hACE2 binding inhibition GMTs of 70% to 130% of reference condition values.

For Omicron BA.5 variant, 18 of 22 samples (81.8%) had total hACE2 binding inhibition titer %GCV <30% (total assay precision for all 22 samples was 22.7% GCV). For inter-assay precision, 20 of 22 samples (90.9%) had %GCV <30%, and 19 of 22 samples (86.4%) had intra-assay %GCV <30%. Incubation with SARS-CoV-2 rS protein before the assay decreased hACE2 binding inhibition titer in 7 of 7 samples, whereas ≤25% of the samples were affected by irrelevant proteins (only 1 of 7 samples [14.2%] were decreased by RSV F at 1.0 ug/mL and 2 of 8 samples [25%] were decreased by Ebola GP at 0.5 ug/mL). Linearity was successfully demonstrated (R^2^ = 0.9965). LLoQ was defined as 10, and ULoQ was determined to be at least 3300. Robustness for incubation time was demonstrated, as 17 of 18 samples (94.4%) at the lower limit of coating time (14 h) and 17 of 18 samples (100%) at the upper limit (48 h) had hACE2 binding inhibition GMTs of 70% to 130% of reference condition values.

In the variant assay validations, we did a separate robustness test of S-protein coating time (up to 72 h of incubation time) to confirm that the minimum and maximum coating times would have appropriate titer percent recovery (within our specifications). Sixteen out of nineteen samples tested (84.2%) had hACE2 inhibition GMTs within the acceptable range (between 80–120% of the reference condition values).

### 3.3. Assay Correlation with other Markers

**Figure 5:**
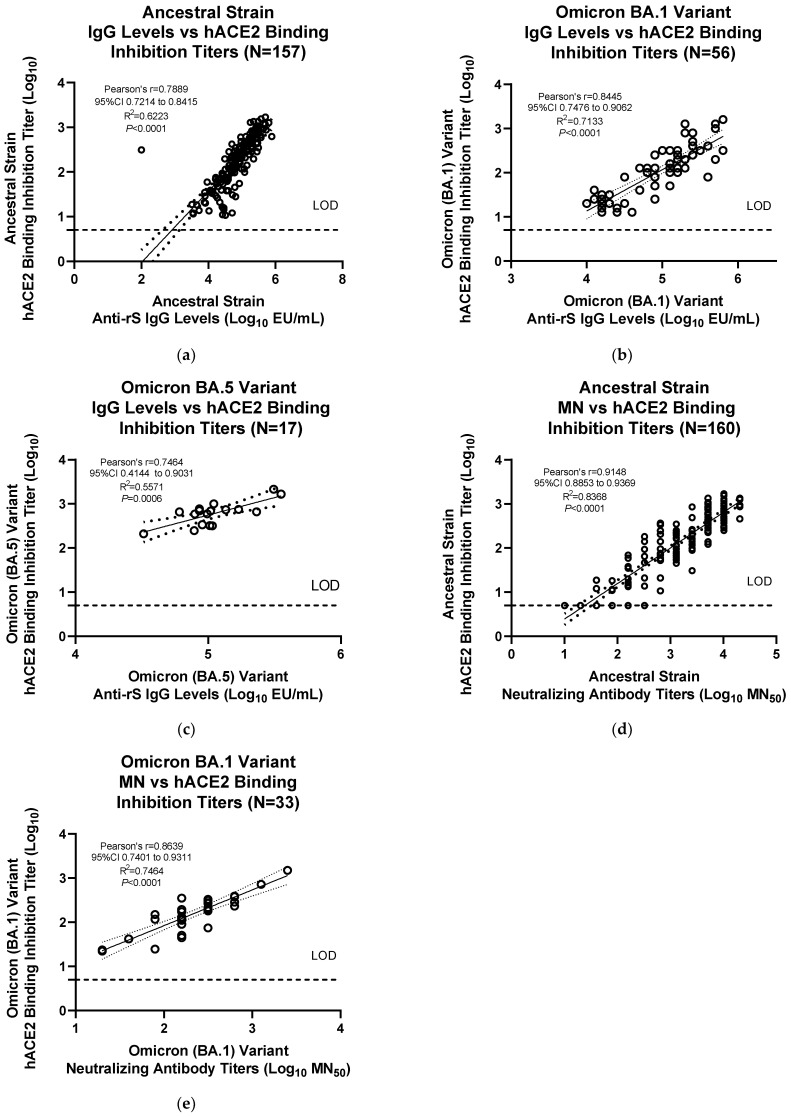
Correlation of ancestral strain and Omicron variant (BA.1) hACE2 binding inhibition with anti-rS IgG levels and neutralizing antibody titers. (**a**–**c**) Detection of anti-S IgG antibodies was performed, as previously described [18]. Linear regression analysis was performed after excluding the hACE2 binding inhibition values at LOD (for **a**,**b**) to compare results from the IgG assay and the hACE2 binding inhibition assay for (**a**) ancestral strain, (**b**) Omicron BA.1 variant, or (**c**) Omicron BA.5 variant. (**d**,**e**) Detection of neutralizing antibodies was performed, as previously described [18]. Linear regression analysis was performed to compare results from the microneutralization assay and the hACE2 binding inhibition assay for (**d**) ancestral strain or (**e**) Omicron BA.1 variant. Serum sample data were from Novavax clinical trials. LOD—limit of detection; Log_10_—logarithm with base 10; MN_50_—50% microneutralization.

The results from the hACE2 assay were significantly correlated with anti-rS IgG levels for the ancestral strain (Pearson’s *r* = 0.846, R^2^ = 0.7157, *p* < 0.0001) and the Omicron BA.1 variant (Pearson’s *r* = 0.8626, R^2^ = 0.7442, *p* < 0.0001) (Figure S1a,b). Correlations without the data points at LOD values for the hACE2 binding inhibition assay demonstrated similar significant correlations with anti-rS IgG levels for the ancestral strain (Pearson’s *r* = 0.789, R^2^ = 0.6223, *p* < 0.0001) and the Omicron BA.1 variant (Pearson’s *r* = 0.8445, R^2^ = 0.7113, *p* < 0.0001) (Figure 5a,b). hACE2 binding inhibition for Omicron BA.5 variant also showed a similar significant correlation with anti-rS IgG assay data (Pearson’s *r* = 0.7464, R^2^ = 0.5571, *p* < 0.0006) (Figure 5c). The results from the hACE2 assay were also significantly correlated with neutralizing antibody titers for the ancestral strain (Pearson’s *r* = 0.9148, R^2^ = 0.8368, *p* < 0.0001) and the Omicron BA.1 variant (Pearson’s *r* = 0.8639, R^2^ = 0.7464, *p* < 0.0001) (Figure 5d,e).

## 4. Discussion

This manuscript describes development and validation of a rapid, high-throughput hACE2 binding inhibition assay, which will be useful for evaluating immunogenicity of COVID-19 vaccines. For ancestral strain, this assay showed acceptable precision, specificity, selectivity, linearity, and robustness (incubation time). Free hemoglobin and lipid (triglyceride) matrix had minimal interference, and sample stability was preserved up to 8 freeze/thaw cycles or up to 24 months in freezer storage.

The assay was also adapted for use with the Delta and Omicron BA.1/BA.5 variants and showed similar results for validation parameters and QCs as for ancestral strain. The hACE2 binding inhibition assay results correlated significantly with results from anti-rS IgG and neutralizing antibody titer assays, for both ancestral strain and the Omicron BA.1/BA.5 variant. These results suggest that the assay will be useful for variant strain immunogenicity assessments in addition to those for ancestral strain, a critical benefit as vaccines are currently being adapted to emerging variants.

Currently, some ACE2 binding inhibition assay kits with a variety of assay end points (such as time-resolved fluorescence energy transfer, chemiluminescence, and colorimetric assays) are commercially available [19]. However, this is the first published report describing the validated ACE2 binding inhibition assay for multiple SARS-CoV-2 variants, including Omicron BA.5 variant. Although our assay method is similar to a previously published ACE2-RBD S-protein binding method [13] in general, there are significant differences. Zhang et al. used hACE2 coating on the plates, whereas our method uses the full-length rS (relevant vaccine antigen) including transmembrane domain to coat plates for identifying the hACE2/S binding inhibition. Use of intact (full-length rS) S-protein instead of RBD [13] might enable more sensitive and comprehensive detection of hACE2 binding inhibition.

In addition, we have performed complete validation of the hACE2 binding inhibition assay including intra-assay and inter-assay precision, linearity, LLoQ, matrix effects, and freeze/thaw effects. Comprehensive understanding of these assay parameters is very important for evaluation of clinical samples and reliable assay performance. Validation of our assay against additional SARS-CoV-2 variants (Delta and Omicron BA.1/BA.5) is an additional element of novelty described in the current manuscript. Because of the newly emerging variants of SARS-CoV-2, validated assays against SARS-CoV-2 variants (especially Omicron) can help in profiling the immune response generated by both prototype and bivalent SARS-CoV-2 booster vaccines.

Because of the limited throughput and turnaround time of assays associated with biosafety level 3 (BSL-3) procedures, the hACE2 binding inhibition assay conducted in BSL-2 laboratories can add significant value. This procedural difference can also result in lower assay costs for hACE2 binding assays. As the hACE2 binding inhibition assay is an in vitro assay (without the need for culturing cells) in contrast to the infectious SARS-CoV-2 microneutralization assay, it can also simplify the assay procedure and provide faster assay data on clinical trial samples. Robust correlation of hACE2 binding inhibition with microneutralization assay using infectious SARS-CoV-2 assay (conducted in BSL-3) for both prototype and Omicron BA.1 variant demonstrates the utility of hACE2 binding inhibition as a surrogate for BSL-3–based infectious SARS-CoV-2 neutralization assay.

A possible limitation of this work is the lower assay value range of the hACE2 binding inhibition assay compared with that of the anti-rS IgG enzyme-linked immunosorbent assays. This might be because of a small subset of binding antibodies being able to block the interaction of S-protein and hACE2 receptor protein. In contrast, the anti-rS IgG assay detects IgG binding to all surfaces of the rS protein. Despite this limitation, in our opinion it may not be a significant problem, because the hACE2 binding inhibition titers correlate very well with the other immunogenicity measures. Pre-dilution of samples was not required but could possibly expand the assay detection range. Other assay readouts such as luminescence might enhance the assay’s dynamic range. This article demonstrates that hACE2 binding inhibition does correlate significantly with both anti-rS IgG levels and neutralizing antibody titers. These two common immunogenicity biomarkers are shown to correlate with vaccine efficacy in findings from other studies [7]. Additionally, the assay has only been validated for ancestral strain, Delta, and Omicron BA.1/BA.5 variants so far. Emerging variants such as Omicron have shown enhanced immune-evasion properties [20,21], suggesting that new variants could show changes in hACE2 binding by S protein (the major target of current vaccines). However, the assay demonstrated utility for ancestral as well as Omicron BA.1 and BA.5 strains, suggesting that this assay will be useful for the currently circulating/emerging variants. At present, we are evaluating the utility of hACE2 binding inhibition as a correlate of protection for vaccine efficacy.

## 5. Conclusions

The hACE2 binding inhibition assay developed and validated in this work is a rapid, high-throughput assay for evaluation of vaccine immunogenicity, without requiring additional biocontainment measures (BSL-3). Results from the hACE2 binding inhibition assay correlated significantly with other clinical vaccine immunogenicity biomarkers (including microneutralization titers and anti-rS IgG levels). Additionally, the hACE2 binding inhibition assay has the potential to be a surrogate of protection, as it can the measure blocking of the binding of S protein to the receptor hACE2. This novel assay can assist with future work on COVID-19 correlates of protection and vaccine development against new variants.

## Figures and Tables

**Table 1:** hACE2 binding inhibition assay ancestral strain precision (intra-assay and inter-assay precision).

Sample	Sample ID	hACE2 Binding Inhibition Titer (GMT)	Inter-Assay %GCV	Intra-Assay %GCV	Total %GCV
1	HQC	158.5	8.2	6.5	10.5
2	LQC	37.5	8.3	7.4	11.1
3	MQC	66.6	8.0	6.9	10.5
4	NC	5.0	0.0	0.0	0.0
5	Serum #3	81.1	15.3	16.1	22.3
6	Serum #1	91.3	18.8	7.3	20.2
7	Serum #2	62.4	7.4	8.5	11.3
8	Serum #12	11.0	13.0	12.7	18.2
9	Serum #13	115.5	11.4	6.2	13.0
10	Serum #14	22.3	6.2	7.3	9.6
11	Serum #15	170.3	7.6	9.2	11.9
12	Serum #16	54.7	11.5	11.3	16.2
13	Serum #17	93.7	10.2	6.7	12.3
14	Serum #18	22.4	8.2	8.8	12.1
15	Serum #19	61.6	10.3	7.6	12.8
16	Serum #20	18.0	4.3	10.2	11.1
17	Serum #21	26.9	12.3	8.3	14.9
18	Serum #22	27.9	8.1	7.4	11.0
19	Serum #23	62.3	7.3	8.6	11.3
20	Serum #24	13.6	12.2	12.6	17.6

%GCV—percent geometric coefficient of variation; GMT—geometric mean titer; hACE2—human angiotensin-converting enzyme 2; HQC—high quality control; ID—identification; LQC—low quality control; MQC—mid quality control; NC—negative control.

**Table 2:** hACE2 binding inhibition assay ancestral strain assay specificity and selectivity.

**Assay Specificity**							
**Sample ID**	**Assay Buffer**	**Incubated with** **S Protein**		**Incubated with** **RSV F Protein**		**Incubated with** **Ebola GP**	
	**GMT**	**GMT**	**% Reduction**	**GMT**	**% Reduction**	**GMT**	**% Reduction**
Serum #45	175.5	31.4	82.1	183.1	−4.3	181.6	−3.5
Serum #17	85.8	21.1	75.4	79.3	7.6	92.6	−7.8
Serum #13	102.1	26.4	74.1	99.8	2.3	111.4	−9.0
Serum #22	24.7	5.0	79.7	26.8	−8.5	26.5	−7.5
Serum #16	58.8	13.1	77.6	63.3	−7.7	56.5	3.9
**Assay Selectivity**							
**Sample ID**	**Sample #**	**hACE2 Binding Inhibition Titer**		**Anti-A/Singapore ^a^ HAI GMT**		**Anti-A/Michigan ^b^ HAI GMT**	
Serum #39 ^c^	1	<10		20 (Day 0)		40 (Day 0)	
Serum #40 ^c^	2	<10		2560 (Day 21)		2560 (Day 21)	
Serum #41 ^c^	3	<10		20 (Day 0)		40 (Day 0)	
Serum #42 ^c^	4	<10		2560 (Day 21)		40 (Day 21)	
Serum #43 ^c^	5	<10		40 (Day 0)		40 (Day 0)	
Serum #44 ^c^	6	<10		2560 (Day 21)		2560 (Day 21)	
Serum #25	7	<10		640		151	
Serum #26	8	<10		523		302	
Serum #27	9	<10		95		80	
Serum #28	10	<10		N/A		142	
Serum #29	11	<10		N/A		36	
Serum #30	12	<10		N/A		170	
Serum #31	13	<10		N/A		640	

^a^ Influenza virus A/Singapore/INFIMH-16-0019/2016; ^b^ Influenza virus A/Michigan/45/2015; ^c^ These samples are from 3 pairs of samples from 3 participants in the influenza vaccine trial; GP—glycoprotein; HAI—hemagglutination inhibition; RSV—respiratory syncytial virus.

**Table 3:** hACE2 binding inhibition assay ancestral strain linearity.

Sample	Parameter	Estimate	95% LCL	95% UCL
19-2021-09/19-2021-11	Slope	0.974	0.947	1.001
	Intercept	0.061	−0.002	0.124
	Residual Variability (% GSD)	0.027 (6.3%)	N/A	
	R^2^	0.999	N/A	
19-2020-10/19-2020-12	Slope	0.984	0.959	1.009
	Intercept	0.085	0.027	0.143
	Residual Variability (% GSD)	0.025 (5.8%)	N/A	
	R^2^	0.999	N/A	

GSD—geometric standard deviation; LCL—lower confidence limit; N/A—not applicable; UCL—upper confidence limit.

**Table 4:** hACE2 Binding Inhibition Assay Ancestral Strain Heme and Lipid Matrix Effects.

**Heme Matrix Effects**					
**Sample**	**Control**	**50% Hemolyzed**		**25% Hemolyzed**	
	**Inhibition** **GMT**	**Inhibition** **GMT**	**% Recovery**	**Inhibition** **GMT**	**% Recovery**
Serum #62	72.1	63.2	87.7	54.7	75.9
Serum #63	16.2	15.7	96.9	16.7	103.1
Serum #64	106.1	118.0	111.2	136.4	128.6
Serum #65	142.6	133.8	93.8	118.3	83.0
Serum #66	42.4	42.6	100.5	41.5	97.9
Serum #67	<10	<10	N/A	<10	N/A
**Lipid Matrix Effects**					
**Sample**	**Control**	**5.0 mg/mL Triglycerides**		**2.5 mg/mL Triglycerides**	
	**Inhibition** **GMT**	**Inhibition** **GMT**	**% Recovery**	**Inhibition** **GMT**	**% Recovery**
Serum #62	72.1	61.5	85.3	71.1	98.6
Serum #63	16.2	21.7	134.0	20.2	124.7
Serum #64	106.1	125.2	118.0	108.8	102.5
Serum #65	142.6	118.6	83.2	111.2	78.0
Serum #66	42.4	44.1	104.0	42	99.1
Serum #67	<10	<10	N/A	<10	N/A

GMT—geometric mean titer.

## Data Availability

Requests for the data presented in this study will be considered by the corresponding author. The data are not publicly available because of proprietary subject and sample information.

## Supplementary Materials

The following supporting information can be downloaded at https://www.mdpi.com/article/10.3390/microorganisms11020368/s1: Table S1: Assay robustness—lower incubation time limit results; Table S2: Assay robustness—upper incubation time limit results; and Figure S1. Correlation of ancestral strain and Omicron variant (BA.1) hACE2 binding inhibition with anti-rS IgG.
