# Supplementary material for: Severe Acute Respiratory Syndrome Coronavirus 2 Receptor (Human Angiotensin-Converting Enzyme 2) Binding Inhibition Assay: A Rapid, High-Throughput Assay Useful for Vaccine Immunogenicity Evaluation
Source: Microorganisms. 2023 Feb 1;11(2):368. doi: 10.3390/microorganisms11020368 (PMC9965183; doi:10.3390/microorganisms11020368)
Supplement: Supplementary file 1 [file microorganisms-11-00368-s001.zip › microorganisms-2101750-supplementary.pdf]

microorganisms

MDPI

Supplement to "Severe Acute Respiratory Syndrome Coronavirus 2 Receptor (Human Angiotensin-Converting Enzyme 2) Binding Inhibition Assay: A Rapid, High-Throughput Assay Useful for Vaccine Immunogenicity Evaluation"

Table S1. Assay robustness - lower incubation time limit results

Sample #	Sample ID	Reference Condition Titer	Lower Incubation Time Limit		% Recovery	% Difference
			Titer[a]	GMT		
1	HQC	158.5	148	141.6	89.3	-10.7
			135.4			
2	MQC	66.6	61.4	60.9	60.9	91.5
			60.5			
3	LQC	37.6	35.8	35.6	35.6	94.7
			35.4			
4	NC	5	5	5	5	100
			5			
5	Serum #12	11	8.7	8.5	8.5	77.7
			8.4			
6	Serum #13	115.5	101.5	102.7	102.7	88.9
			103.9			
7	Serum #14	22.3	19.5	19	19	85.4
			18.6			
8	Serum #15	170.3	149.3	157.5	157.5	92.5
			166.2			
9	Serum #16	54.7	48.6	47.4	47.4	86.6
			46.2			
10	Serum #17	93.7	76	76.7	76.7	81.9

			77.4			
11	Serum #18	22.4	21.8	21	21	93.9
			20.3			
12	Serum #19	61.6	50.3	51.3	51.3	83.3
			52.4			
13	Serum #20	18	19.1	17.7	17.7	98.3
			16.4			
14	Serum #21	26.9	26.2	26.3	26.3	98
			26.5			
15	Serum #22	27.9	26.1	26.3	26.3	94.4
			26.6			
16	Serum #23	62.3	52.3	55.1	55.1	88.4
			58			
17	Serum #24	13.6	14.9	14	14	103.1
			13.2			
18	Serum #1	91.4	91.5	88.4	88.4	96.8
			85.5			
19	Serum #2	62.4	54.9	58.5	58.5	93.7
			62.3			
20	Serum #3	81.1	88.3	90.2	90.2	111.3
			92.2			

[a]Samples were tested in duplicate in each assay, so 2 titer values are shown for each sample.
Numbers in italics indicate values outside of acceptable range.
GMT—geometric mean titer; HQC—high quality control; ID—identification; LQC—low quality control; MQC—mid quality control; NC—negative control.

Table S2. Assay robustness - upper incubation time limit results

Sample #	Sample ID	Reference Condition Titer	Upper Incubation Time Limit		% Recovery	% Difference
			Titer[a]	GMT		
1	HQC	158.5	149.4	154	97.1	-2.9
			158.7			
2	MQC	66.6	64.4	62.9	94.5	-5.5
			61.5			
3	LQC	37.6	39	39.3	104.5	4.5
			39.6			
4	NC	5	5	5	100	0
			5			
5	Serum #12	11	10.3	10.8	98.5	-1.5
			11.4			
6	Serum #13	115.5	114.4	113.5	98.3	-1.7
			112.6			
7	Serum #14	22.3	23.8	24.4	109.6	9.6
			25.1			
8	Serum #15	170.3	161.7	175.6	103.1	3.1
			190.8			
9	Serum #16	54.7	59	60.2	110.1	10.1
			61.5			
10	Serum #17	93.7	95.5	94.6	101	1
			93.7			
11	Serum #18	22.4	27	*27.4*	*122.5*	*22.5*
			27.9			

12	Serum #19	61.6	63.6<br>86.2	*74*	*120.2*	*20.2*
13	Serum #20	18	19.1<br>21.2	20.1	111.8	11.8
14	Serum #21	26.9	28.7<br>31.5	30.1	111.8	11.8
15	Serum #22	27.9	31<br>30	30.5	109.3	9.3
16	Serum #23	62.3	61<br>70.4	65.5	105.2	5.2
17	Serum #24	13.6	16.3<br>15.9	16.1	118.4	18.4
18	Serum #1	91.4	97.6<br>89.7	93.6	102.4	2.4
19	Serum #2	62.4	61<br>61.5	61.2	98.2	-1.8
20	Serum #3	81.1	117.9<br>151.8	133.8	*165*	*65*

[a] Samples were tested in duplicate in each assay, so 2 titer values are shown for each sample.
Numbers in italics indicate values outside of acceptable range.
GMT—geometric mean titer; HQC—high quality control; ID—identification; LQC—low quality control; MQC—mid quality control; NC—negative control.

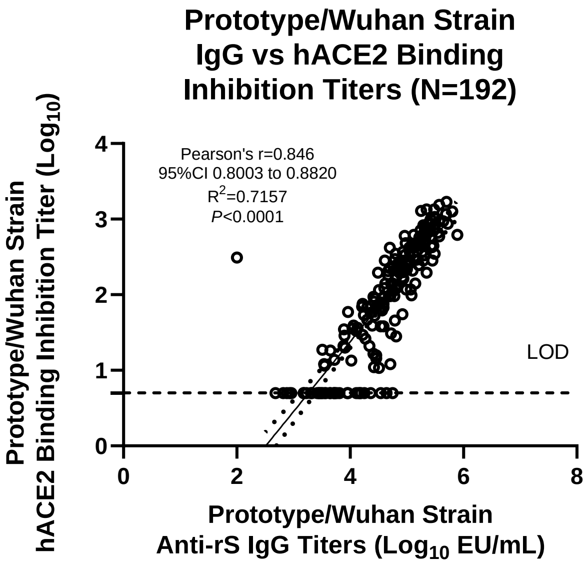

(a)

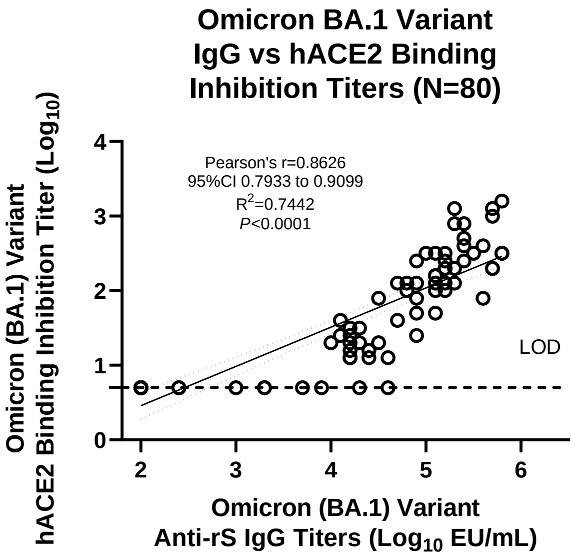

(b)

Figure S1. Correlation of prototype/Wuhan strain and Omicron variant (BA.1) hACE2 binding inhibition with anti-rS IgG. (**a-b**) Detection of anti-rS IgG antibodies was performed as previously described in Formica et al. 2021 [18]. Linear regression analysis was performed with all the hACE2 binding inhibition results, including values at LOD, to compare results from the IgG assay and the hACE2 binding inhibition assay for (**a**) prototype/Wuhan strain or (**b**) Omicron BA.1 variant. hACE2—human angiotensin-converting enzyme 2; IgG—immunoglobulin G; LOD— limit of detection; Log_{10}—logarithm with base 10; rS—recombinant spike.
